# Virtual Reality–Delivered Exposure for Contamination Concerns in Adults With Obsessive-Compulsive Symptoms: Single-Arm Pilot Study

**DOI:** 10.2196/78169

**Published:** 2026-06-23

**Authors:** Anna Caltabiano, Aniruddha Voruganti, Jacqueline Nesi, Georgina Krebs, Taylor Burke, Martina Di Simplicio, Nejra van Zalk

**Affiliations:** 1 Dyson School of Design Engineering, Imperial College London, Exhibition Road, South Kensington, London, SW7 2AZ, United Kingdom, 44 020 7589 5111; 2 Medical Sciences Division, Oxford University Clinical Academic Graduate School, Oxford, United Kingdom; 3 Department of Psychiatry, Warren Alpert Medical School of Brown University, Providence, RI, United States; 4 Department of Clinical, Educational and Health Psychology, University College London, London, United Kingdom; 5 Department of Psychiatry, Harvard Medical School, Massachusetts General Hospital, Boston, MA, United States; 6 Division of Psychiatry, Department of Brain Sciences, Imperial College London, London, United Kingdom

**Keywords:** virtual reality, contamination, exposure, obsessive-compulsive disorder, subclinical, subthreshold

## Abstract

**Background:**

Exposure and response prevention is a first-line intervention for obsessive-compulsive disorder (OCD), yet many individuals with contamination concerns do not access care. Virtual reality exposure–based therapy (VRET) may improve scalability and acceptability.

**Objective:**

This pilot study evaluated the feasibility and acceptability of a standardized single-session VRET protocol targeting contamination concerns and whether it elicited within-session anxiety and exploratory contamination symptom change at 1-month follow-up.

**Methods:**

We conducted a single-arm pilot study in adults with elevated contamination concerns and no formal OCD diagnosis, recruited via convenience sampling. Participants completed a baseline survey, an in-laboratory VRET session using a standardized virtual public toilet environment, and a follow-up survey. Outcomes included momentary anxiety (Subjective Units of Distress Scale) during exposure, affect (positive and negative affect schedule) across time points, and contamination symptoms (Obsessive-Compulsive Inventory–Revised contamination subscale) at baseline and follow-up. Usability (System Usability Scale) and VR sickness were also assessed. Within-session outcomes used repeated-measures ANOVA or Friedman tests; symptom change used paired *t* tests (α=.05); point estimates include 95% CIs. Missing data were addressed using multiple imputation (random forest; m=5); 37.5% of participants did not complete the follow-up survey (overall missingness: 5.47%).

**Results:**

Sixteen participants were included (aged 18‐32 years). Anxiety increased during exposure tasks and decreased after virtual hand washing in both trials (Exposure 1 Friedman Test: *χ*²_3_=28.56; *P*<.001; W=0.6); Exposure 2 repeated measures ANOVA: *F*_1.85, 27.81_=5.35; *P*=.01; Greenhouse-Geisser corrected=0.058. Negative and positive affect both changed significantly across time points (negative affect: Friedman Test: *χ*²_3_=13.76; *P*=.003; W=0.29 and positive affect: repeated measure ANOVA: *F*_3, 45_=4.60‐4.71; *P*=.006-.007; Greenhouse-Geisser corrected=0.07‐0.073). Contamination symptoms did not significantly change from baseline to follow-up (mean change 1.30, SD 3.39; 95% CI –0.36 to 2.96; *P*=.12). Usability was adequate (System Usability Scale mean 69.5, 95% CI 62.79-76.21).

**Conclusions:**

This study is among the first to systematically evaluate a standardized single-session contamination-focused VRET protocol in adults with elevated contamination concerns who did not meet diagnostic criteria for OCD, a subthreshold population underrepresented in prior VRET research, which has focused on clinically diagnosed samples and multisession protocols. The protocol proved feasible and acceptable, eliciting within-session anxiety and providing benchmarks in existing clinical literature. Contamination symptom change at 1-month follow-up was not statistically significant, and the effect size estimate was sensitive to the missingness assumption, underscoring the need for adequately powered multisession designs in future trials. The usability profile and VR sickness levels that did not prevent session completion suggest self-administered or minimally supervised delivery warrants evaluation, with implications for scalable early intervention in individuals with elevated contamination concerns who have not accessed formal treatment.

## Introduction

### Background

Obsessive-compulsive disorder (OCD) is a disorder characterized by intrusive thoughts, images, or urges, which are distressing and often result in repetitive physical or mental acts that the individual feels driven to perform to temporarily alleviate their distress [1]. It is associated with income loss, decreased quality of life, and substantial economic burden [2,3]. According to the National Comorbidity Survey Replication, the lifetime prevalence of OCD is approximately 2.3% [4], though this is thought to be an underestimate due to the stigma often experienced by those with OCD, leading to underreporting of symptoms [5]. The most widespread dimension experienced by people with OCD concerns contamination/washing, with up to 50% of people with OCD reporting contamination-related symptoms [1]. These fears often manifest as persistent and intrusive thoughts about being contaminated by germs, dirt, or other harmful substances [1], and typically drive compulsive washing or cleaning behaviors, which the individual performs repeatedly to reduce anxiety and prevent perceived contamination [6]. This cycle of obsessive fear and ritualistic behavior can significantly interfere with daily functioning and overall quality of life.

The prevalence of contamination-related OCD has increased since the COVID-19 pandemic [7]. Similarly, symptoms related to fear of contamination have also increased in nondiagnosed populations [7], although it remains unclear whether they will remain elevated. People with mild or undiagnosed OCD symptoms are often overlooked in treatment and research, despite evidence demonstrating that conditions falling below diagnostic cut-offs for anxiety-related disorders still have considerable clinical significance, and these early signs can often predict meeting full diagnostic criteria for OCD in the future [8-10]. This could be due to reasons such as not meeting the criteria for severity of symptoms, recurrence, presence of obsessions without compulsions, insufficient time consumption, or not causing significant day-to-day impairment. Undiagnosed individuals may include those who meet the criteria for diagnosis but have not sought or had the opportunity to receive a diagnosis, as well as those who do not meet diagnostic criteria (also called subclinical or subdiagnostic symptoms, but described in this paper as not meeting diagnostic criteria). As symptoms occur on a continuum [11], research into symptoms that do not meet diagnostic criteria is of clinical value. Symptoms of anxiety-related disorders, including OCD, exhibit significant comorbidity and sequential comorbidity with each other, even at levels below the clinical threshold [12,13]. Gaining a deeper understanding of OCD symptoms below the diagnostic threshold could pave the way for early intervention, reducing the risk of progression to clinical diagnosis.

### Traditional Treatment

The traditional first-line gold-standard treatment for OCD is cognitive-behavioral therapy incorporating exposure and response prevention [14], which is considered a key active ingredient of this approach. Exposure-based therapy involves confronting a fear-provoking stimulus in a controlled environment, enabling anxiety habituation and/or belief disconfirmation. Its effectiveness as an intervention for fear and anxiety has been well-documented [15,16]. Nevertheless, exposure-based therapy is not for everyone. The majority of people with OCD cannot access evidence-based treatment [17]. Even among those with access to treatment, delays are common, with the average untreated illness lasting over 7 years [18]. These delays are concerning, as longer durations of untreated illness are associated with poorer treatment outcomes [19] and spontaneous remission is rare in OCD [20]. Hurdles to receiving treatment include cost, finding a relevant clinician, and taking time off from school/work for multiple sessions [21]. Thus, new intervention solutions are necessary to effectively address these evolving challenges.

### Virtual Reality–Delivered Exposure-Based Therapy

Digital exposure therapy offers a promising solution to treatment access barriers, providing benefits such as scalability, personalization, and a lower refusal rate compared to traditional exposure-based therapy, suggesting individuals may be more open to it [22-24]. Virtual reality (VR) is an immersive technology in which a participant experiences and engages with a customized virtual environment other than the external physical environment around them [25]. It has been successfully used to deliver therapy for various conditions such as posttraumatic stress disorder, addiction, eating disorders, and acute and chronic pain, as well as for many anxiety-related disorders, such as generalized anxiety disorder, social anxiety disorder, and numerous phobias [26].

Meta-analyses suggest that virtual reality exposure–based therapy (VRET; also known as VRE) is effective for various anxiety-related disorders and is not inferior to traditional in vivo exposure therapy [27,28]. Due to its potential to encourage realistic behavior in the virtual environment, it is believed that the plausibility and sense of physical presence (ie, being physically present and immersed) in the VR environment enhance engagement, making exposure therapy more effective [29]. Additionally, the documented lower refusal rate may be especially beneficial for the treatment of OCD due to its typical high refusal and drop-out rates [30,31]. VRET can be conducted in a clinical setting, administered by a clinician, or self-administered at home. This flexibility enables individuals to receive treatment without necessarily needing to take time off work or school for travel.

Nevertheless, VRET is not without its limitations. Individuals may experience VR sickness (also called cybersickness), which presents as dizziness, nausea, and other similar motion sickness–related symptoms. While accessibility is a challenge for VRET, as VR technology is still in early stages of adoption by the general public, the global VR market is forecast to hit US $103 billion by 2027 [32]. The hope is that advancements will make it increasingly accessible, potentially even more so than traditional treatment. Developing an optimal protocol for its operation and implementation to maximize effectiveness and safety remains a crucial challenge.

A modest but growing literature suggests that virtual reality can be used to provoke obsessive-compulsive-relevant distress and deliver exposure-based procedures, including contamination-focused paradigms [33-36]. Collectively, these reviews suggest increasing support for VR as a tool for symptom provocation and exposure delivery in OCD, while also highlighting heterogeneity in protocols and a need for more standardized approaches and clearer characterization of target populations. Recent systematic reviews and meta-analyses have summarized extended reality (XR)/VR applications for OCD and contamination-related concerns, including both clinical and nonclinical samples [37-40], and randomized studies have compared VR-based exposure and response prevention with in vivo exposure approaches [33,41]. Against this background, there remains limited work examining standardized single-session VR exposure protocols in individuals with elevated contamination concerns who do not meet diagnostic criteria for OCD, despite potential relevance for scalable early intervention and for improving treatment engagement. VR environments designed for exposure-based therapy have been validated in their ability to provoke OCD-relevant distress and compulsive behaviors, yet some findings [34] suggest they may not consistently sustain anxiety levels throughout exposure, which could affect fear extinction processes and optimization of exposure dose. However, an earlier study [35] suggested that repeated VR exposure can reduce contamination-related distress, demonstrating its potential as an intervention. Research in comparisons between VRET and in vivo exposure also indicates that VR can provoke similar anxiety responses during exposure, but protocol standardization and long-term effectiveness remain unresolved [42]. Additionally, while VR has been shown to effectively provoke contamination-related anxiety and disgust, its impact on symptom reduction has not been fully examined, limiting its clinical applicability [36]. These gaps highlight the need for systematic, carefully controlled VRET research that explores its efficacy in anxiety induction, symptom reduction, and participant acceptability.

In addition to this broader evidence base, early studies on VRET for contamination fear [35,36] established proof of concept but faced significant limitations. Variability in session lengths, number of sessions, and VR scenario content hindered protocol standardization, limiting the clinical relevance and reproducibility of findings [35]. Moreover, previous studies [35,36] offered limited exploration of user experience, such as how individuals perceive and respond to VR interventions, usability of the program, or whether the VRET program was deemed acceptable by participants as an intervention.

Prior VR exposure studies for contamination concerns have varied substantially in dose, including session length, number of sessions, and exposure structure, which complicates interpretation and replication across studies and limits the ability to identify which procedural elements are most responsible for observed effects. In the present study, we selected a single-session design to standardize intervention delivery and evaluate the feasibility, acceptability, and anxiety elicitation capacity of a clearly specified VR exposure protocol. This approach also aligns with broader work on single-session interventions that aim to increase scalability and reduce barriers to engagement, while generating preliminary parameter estimates to guide subsequent multisession and controlled trials [43]. The single-session format was therefore intended as an initial, low-burden step to test whether a standardized contamination-focused VR exposure session could reliably induce distress during exposure tasks and be tolerated by participants.

### This Study

To address limitations of prior studies, this small-scale pilot study evaluated the feasibility and acceptability of a standardized single-session VR exposure protocol targeting contamination concerns and characterized within-session distress responding. Symptom outcomes were assessed exploratorily from baseline to 1-month follow-up to inform future controlled trials. This study sought to address the variability found in earlier research by using a standardized protocol with 2 identical exposures within a single session, modeled after Inozu et al [36], Schleider et al [44], and Schleider and Beidas [43], ensuring consistent conditions for all participants and enhancing the reliability of the findings. Additionally, by studying participant experience, including acceptability, VR sickness, and feedback for potential improvements, we aimed to advance understanding of the usability of VRET and inform future intervention design. As no prior studies have focused on this group, we did not formulate specific hypotheses. Instead, we explored the following research questions: (1) Can this single-session VRET intervention effectively induce anxiety to facilitate exposure? (2) How do participants rate their experience of the VRET, and do they find it acceptable as an intervention? (3) What preliminary changes in self-reported contamination symptoms are observed from baseline to 1-month follow-up?

## Methods

### Inclusion and Exclusion Criteria

Participants were recruited via Instagram (Meta) advertisements and posters placed around the main campus of Imperial College London, directing them to an online screening survey on Qualtrics. The advertisements and posters inquired if potential participants found public toilets stressful and invited them to participate in a VR study exploring discomfort around contamination. This recruitment approach reflects convenience sampling of volunteers responding to study advertisements.

A total of 48 individuals opened the online screening survey. Of these, 6 were excluded at screening, primarily due to self-reported use of medication that could affect heart rate or anxiety responses and self-reported current or past treatment for OCD. Forty-two individuals met eligibility criteria and were invited to participate. Eighteen participants scheduled and attended the in-laboratory session.

Participants were required to be 18 years or older English speakers with contamination concerns. To capture a full range of contamination-related anxiety, these concerns were defined as either a score greater than 0 on the contamination subscale of the Obsessive-Compulsive Inventory–Revised (OCI-R [45]) or a response of 3, 4, or 5 (indicating “sometimes,” “often,” or “always”) to at least one of the following questions/statements constructed for the purposes of this study: (1) “I experience anxiety or distress when I see a dirty toilet,” (2) “How often do you find yourself avoiding public toilets due to fear or worry that they might be too dirty?” and (3) “How often do you find yourself unable to use public toilets due to fear or worry that they might be too dirty?”

Exclusion criteria included reporting any condition precluding adherence to the measurement protocol (such as difficulty reading or writing), any uncorrected visual and/or auditory impairment affecting full participation in VR (use of corrective lenses or hearing aids was not a criterion for exclusion), current or past diagnosis of OCD (to target below-threshold OCD symptoms), current or past treatment for OCD (including psychological and pharmacological therapies), presence or history of epilepsy, and history of severe motion sickness or dizziness. Participants were also excluded if they self-reported taking medication at the time of this writing that affected heart rate, such as benzodiazepines. Eligibility and exclusion criteria were assessed entirely by self-report in the screening survey.

### Sampling Procedures

A total of 18 participants signed up and gave informed consent. Data from 2 participants were excluded due to technical difficulties with the VR hardware, leaving 16 participants in the final analysis. Ten of these 16 (62.5%) participants completed the one-month follow-up survey.

### Participant Characteristics

**Figure 1.**
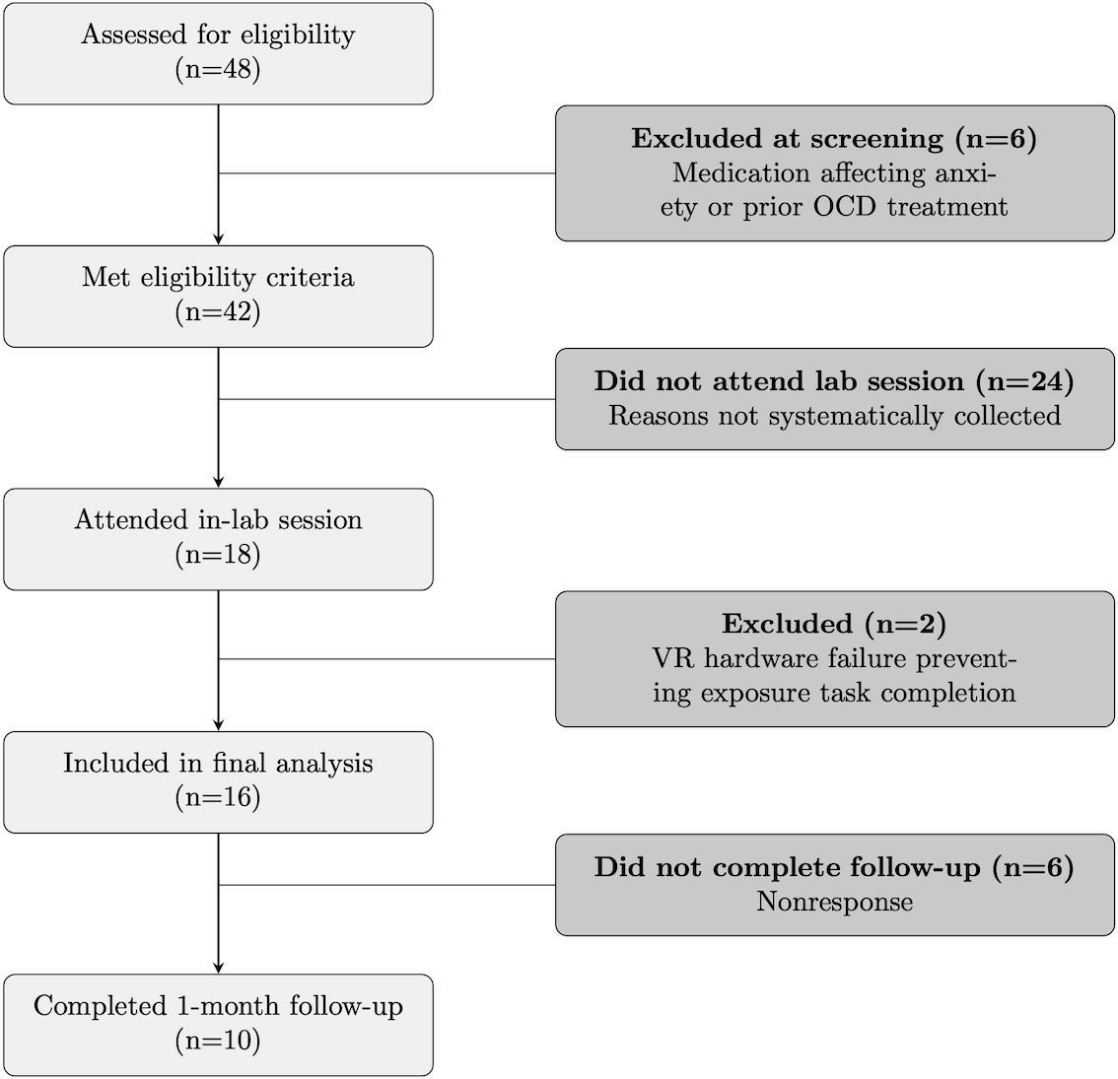
Participant flow diagram for a single-arm, pretest–posttest, single-session virtual reality (VR) exposure pilot study in adults with subthreshold contamination concerns at Imperial College London (London, United Kingdom), February-June 2023. OCD: obsessive-compulsive disorder.

Participant characteristics are summarized in the Results section. The participant flow is illustrated in Figure 1.

### Procedure

We conducted a single-group, pretest-posttest pilot study with a one-month follow-up. Participants who met the inclusion criteria and consented to take part in the study were invited to an in-laboratory session where they were given an online Qualtrics (Qualtrics LLC) survey to collect baseline information on their OCD symptoms and factors that may influence their levels of anxiety (eg, medication taken, exercise today, and illness). Participants were then given a brief information sheet about exposure therapy and read a short script providing the context to the VR scenario they would experience shortly.

Before entering the VR environment, participants were asked to select the skin color of their avatar. This was done to enhance the experience of embodiment in the virtual environment. Participants then experienced 2 identical self-paced VR exposures and viewed a short neutral video between exposure sessions. Virtual hand-washing was included as the concluding element of each exposure trial. This was a deliberate design choice reflecting the ecologically valid sequence of behavior in a public toilet context, as washing hands is a normative response after toilet use, and it was supported by the structure of the Amelia by XR Health (XRHealth) environment. Consistent with the study’s pilot aims, which centered on feasibility, acceptability, and anxiety elicitation rather than delivering a full exposure and response prevention (ERP) protocol, response prevention was not imposed. This design choice is acknowledged as a limitation when interpreting the anxiety reduction data (see Discussion). Participants were asked to rank their anxiety (Subjective Units of Distress Scale [SUDS]; [46]) 4 times during each exposure. They reported on their affect (Positive and Negative Affect Schedule [PANAS]; [47]) before, between, and after these VR sessions. They were then asked about VR presence and plausibility, with additional repeated measures taken on affect. One month later, an online follow-up survey was emailed to participants to assess OCD symptoms, attitudes relating to contamination, and positive and negative affect.

### Ethical Considerations

Ethics approval was granted by the Imperial College Research Governance and Integrity Team (Reference number: 22IC7615). All participants provided informed consent prior to completing any study procedures, and participants could discontinue participation at any time without penalty. Data were collected using Qualtrics and stored in access-controlled systems; data were deidentified prior to analysis and reported only in aggregate form. Participants who completed the in-laboratory session were entered into a raffle to win a £30 (approximately US $36.22; conversion rate: 1 GBP=US $1.2073; February 2023 monthly average) Amazon e-voucher; compensation was not contingent on completion of the one-month follow-up survey. The manuscript figures and materials do not include identifiable images of participants.

### Measures and Covariates

#### Demographics

Participants self-reported age, sex, and whether their gender identity matched their sex assigned at birth. They were also asked to self-report variables that could impact anxiety, including caffeine intake, exercise, medication, and illness. Additional demographics, such as ethnicity and education, were not collected to keep the protocol brief given the low-burden compensation and to minimize collection of potentially identifying information, consistent with ethics review guidance.

#### Psychiatric Diagnoses

Participants self-reported any past or current psychiatric diagnoses. Participants reported contamination-featured OCD symptoms using the Washing subscale of the OCI-R at baseline (α=.83; SE 0.07; 95% CI 0.68-0.97) and at one-month follow-up (α=.59; SE 0.19; 95% CI 0.22-0.95). The OCI-R is a widely used self-report measure of OCD symptoms, comprising 18 items that assess symptoms across 6 subscales. Each item is rated on a 5-point Likert scale ranging from 0 (“not at all”) to 4 (“extremely”). The contamination subscale includes 3 items, with a total score range of 0-12, where higher scores indicate greater contamination-related distress.

#### Anxiety and Affect

Momentary anxiety was measured using the one-item SUDS rated on a scale from 0 to 10, administered verbally during each VR exposure. To measure positive and negative affect, the short form of the PANAS was administered at baseline (positive subscale: α=.82; SE 0.07; 95% CI 0.69-0.95; negative subscale: α=.71; SE 0.11; 95% CI 0.50-0.92), after the first VR exposure (positive subscale: α=.76; SE 0.09; 95% CI 0.58-0.93; negative subscale: SE 0.05; 95% CI 0.58-0.93), after the break between exposure sessions (positive subscale: α=.82; SE 0.07; 95% CI 0.70-0.95; negative subscale: SE 0.04; 95% CI 0.80-0.97), and after the second VR exposure (positive subscale: α=.83; SE 0.06; 95% CI 0.70-0.95; negative subscale: α=.95; SE 0.02; 95% CI 0.91-0.99). The PANAS consists of 2 scales (one for positive affect and one for negative affect), both on a 5-point Likert scale.

#### User Experience and Acceptability

VR presence and plausibility were measured by 2 questions each that were created for this study, adapted from Slater [29]. To measure VR plausibility, we asked participants to compare the virtual environment to (1) a real public toilet and (2) an imagined public toilet environment. The responses are on a 5-point Likert scale, where 1 is much more anxious in VR and 5 is much more anxious in the real or imagined public toilet environment. VR sickness was measured using the Simulator Sickness Questionnaire (SSQ [48]) at baseline (α=.86; SE 0.05; 95% CI 0.76-0.96) and postintervention (α=.77; SE 0.08; 95% CI 0.60-0.93). The SSQ is on a 4-point Likert scale. Acceptability was measured using the System Usability Scale (SUS [49]) on a 5-point Likert scale and an α of .81 (SE 0.07; 95% CI 0.68-0.95), as well as open-ended questions for descriptive analysis, such as “How did you feel while you were taking part in the virtual reality experience?” and “In your opinion, is there anything that could have been done differently to improve the software or create a better experience (in any aspect)?”

### Equipment and Environment

**Figure 2.**
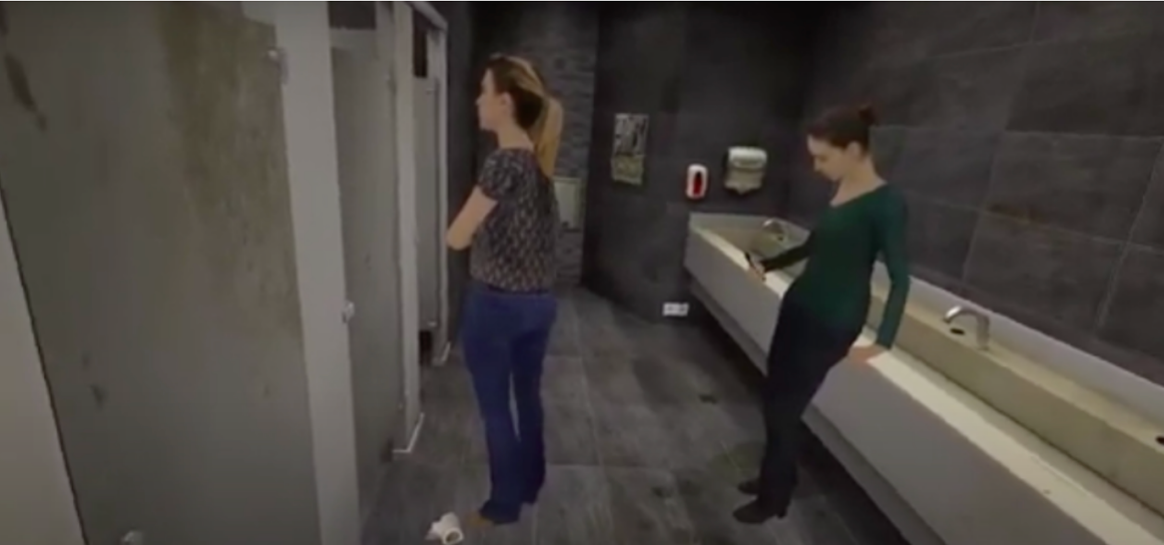
Screenshot of the Amelia by XR Health virtual public toilet environment used in a single-arm, pretest-posttest, single-session virtual reality exposure pilot study in adults with subthreshold contamination concerns at Imperial College London (London, United Kingdom), February-June 2023.

We used Amelia by XR Health’s public-toilet VR environment, which depicts a contaminated public toilet in a subway area (Figure 2). The scenario begins with the participant standing outside the door of a public toilet. The participant sees other individuals by the cubicles and the sink upon entering. The participant is able to enter the cubicle in front of them and interact with the toilet-related elements (eg, toilet lid, toilet paper, and toilet brush) and can sit down on the toilet (the participant can simultaneously sit in a chair in the real world to mirror their position in VR). The participant is then placed in front of the sink and able to wash their hands in the virtual scenario. The software was run on PICO G2 (Pico Interactive Inc) head-mounted display VR headsets.

The VR environments are controlled through Amelia by XR Health’s online platform, where the following actions can be taken: open the entrance door, open the cubicle door, touch the wall while standing inside the cubicle, lower the toilet lid, flush the toilet, touch the wall while sitting on the toilet, take toilet paper, touch the toilet brush, and wash hands. In this study, participants completed the actions in the given order at their own pace. When asked to sit on the virtual toilet, a real-life chair was provided to heighten the sense of immersiveness in the virtual environment.

### Sample Size, Power, and Precision

In this study, feasibility refers to whether the protocol could be successfully conducted, encompassing recruitment, retention, protocol adherence, and data completion, while acceptability refers to participant experience and willingness to engage with the intervention, indexed by usability ratings, VR sickness, and qualitative feedback. These constructs are treated as distinct throughout, consistent with pilot study reporting conventions [50,51]. This study was designed as a pilot to evaluate feasibility and acceptability of a standardized single-session VR exposure protocol and to characterize within-session distress responding. As a planning benchmark, prior VRET studies reported effect sizes ranging from Hedges *g*=0.88-1.08 [27,28], and a sample of 14-20 participants would be needed to detect effects in this range at α=.05 and power=0.95 using a 2-tailed Wilcoxon signed-rank test. However, these effect sizes derive from multi-session interventions in clinical samples and may not generalize to a single-session, subthreshold pilot; accordingly, this calculation should be understood as an approximate recruitment benchmark rather than a definitive power analysis for detecting symptom change in this design. The study was not powered to detect small-to-moderate changes in contamination symptoms.

### Data Analysis

Pilot studies are commonly sized to support preliminary estimation (eg, variability and CIs) and to identify procedural issues relevant to scaling, rather than to provide definitive hypothesis tests of clinical efficacy [52]. Accordingly, we aimed to recruit approximately 15-30 participants, which is consistent with common recommendations for feasibility and pilot work and sufficient to yield stable estimates of feasibility and acceptability outcomes and to inform the design of future randomized trials [53].

The study was preregistered on AsPredicted (AsPredicted.org) [54]. After preregistration, we refined the analytic focus to prioritize outcomes directly related to the study’s primary aims of feasibility, acceptability, and within-session distress responding, as well as exploratory symptom change at follow-up. Accordingly, several secondary measures that were collected (Patient-Reported Outcomes Measurement Information System, Work and Social Adjustment Scale, and Distress Tolerance Scale) were not analyzed in the present report because they were not central to these aims and would substantially expand the number of analyses in a small pilot sample. The Distress Tolerance Scale was included as a trait-oriented measure and was not designed to assess within-session change during exposure, which was the focus of the current analyses. Physiological data were also collected but were not analyzed in this study.

Although the preregistration noted an expected pattern of distress activation and reduction during exposure, the present report is framed around research questions consistent with the study’s pilot aims rather than formal hypothesis testing. Two participants were excluded due to VR hardware issues that prevented completion of the exposure task and resulted in incomplete data.

Statistical analyses were performed in R (R Core Team). Data were analyzed to explore symptom outcomes, participant experience, and potential outcome predictors. To examine symptom outcomes, statistical analyses were selected based on normality assessments (Shapiro-Wilk tests). Repeated measures ANOVAs were conducted for variables meeting assumptions of normality (positive affect), while Friedman tests, a nonparametric alternative, were used for variables violating these assumptions (negative affect and anxiety levels). For within-session SUDS ratings, normality was evaluated separately for each exposure trial; Exposure 1 SUDS violated distributional assumptions and was analyzed using the Friedman test, whereas Exposure 2 SUDS met normality assumptions and was analyzed using a repeated-measures ANOVA (Greenhouse-Geisser corrected as needed). Given the pilot sample and the similarity of the 2 exposure trials, we interpret inferential results within each trial descriptively and avoid overinterpreting differences in statistical significance between trials. Paired *t* tests were additionally performed to compare pre- and postintervention scores for contamination symptoms, which met normality criteria. Results that were identical across the 5 imputed datasets (ie, did not use any imputed data) were reported based on a single calculation from one dataset, while results based on missing data were pooled using Rubin’s rules if appropriate. For Friedman and ANOVA, a range was reported to show variation in results across datasets. Effect size calculations were conducted to investigate the magnitude of changes. To examine participant experience, descriptive data for virtual sense of presence and plausibility, descriptive data for intervention acceptability, frequency of item endorsement on the SUS, and open-answer participant feedback for areas of intervention improvement were examined. As an exploratory analysis, we examined whether baseline contamination severity (OCI-R contamination subscale) was associated with peak subjective distress during the VR protocol, indexed by the SUDS rating collected at the end of the first VR exposure, prior to virtual handwashing (following the highest-intensity contact task), which was expected to capture the highest distress response relative to the second exposure. Mixed effects models were considered but not emphasized due to limited precision in a small pilot sample.

Overall cell-level missingness was 5.47%. However, 6 out of 16 (37.5%) participants did not complete the 1-month follow-up survey, resulting in missing follow-up symptom data. Missingness was primarily due to follow-up nonresponse and occasional skipped questionnaire items. Because missingness could not be assumed, particularly for follow-up nonresponse, we addressed missing values using multiple imputation with chained equations (random forest) under a missing-at-random assumption conditional on observed variables included in the imputation model. Five imputed datasets were generated; estimates were pooled using Rubin’s rules when appropriate, and for omnibus repeated-measures tests we report the range of results across imputations. Statistical significance was defined at *P*<.05.

Little’s missing completely at random test was not feasible due to the perfectly monotone missing data pattern; the same 6 participants were missing all follow-up variables, as none of them returned the one-month follow-up survey. This rendered the covariance matrix required by the test rank-deficient. As an alternative diagnostic, we compared completers and noncompleters on baseline contamination scores and baseline negative affect using Mann-Whitney *U* tests. No statistically significant differences were found (contamination: W=39, *P*=.35; negative affect: W=37.5, *P*=.44), providing limited but consistent evidence that follow-up nonresponse was not strongly predicted by baseline symptom severity or affect.

## Results

### Overview

The sample comprised 6 females, 9 males, and 1 individual who preferred not to say; 87.5% of participants reported a gender identity that matched their sex assigned at birth. Participants were aged 18‐32 (mean 21.75, SD 3.86) years.

**Figure 3.**
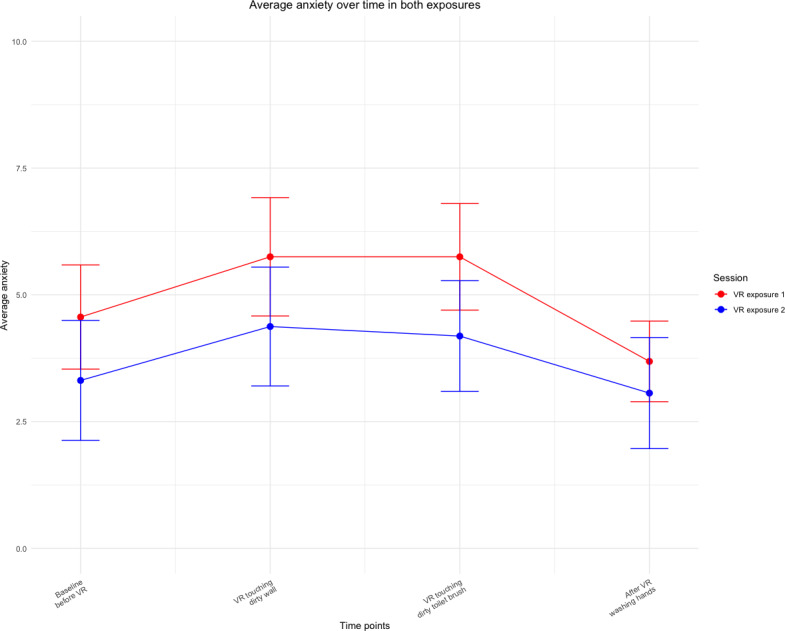
Mean momentary anxiety ratings (Subjective Units of Distress Scale [SUDS]) across standardized time points during 2 virtual reality public toilet exposure trials in a single-arm, pretest-posttest, single-session pilot study in adults with subthreshold contamination concerns at Imperial College London (London, United Kingdom), February-June 2023. SUDS scores range from 0 to 10, with higher scores indicating greater anxiety. Error bars represent 95% CIs.

Baseline contamination symptoms (as measured by the contamination subscale on the OCI-R) ranged from 0 to 11 (mean 5.75, SD 3.51), out of a maximum score of 12. One participant scored 0 on the OCI-R contamination subscale at baseline but met study eligibility criteria based on screening endorsement of contamination concerns and reported distress during the VR exposure tasks. Figure 3 presents responses from the 12 participants who completed the screening items. Responses to the question “How often do you find yourself avoiding public toilets for fear or worry that they might be too dirty?” were distributed as follows: always (n=1), often (n=7), sometimes (n=2), rarely (n=1), and never (n=1). Responses to the question “How often do you find yourself unable to use public toilets for fear or worry that they might be too dirty?” were always (n=0), often (n=5), sometimes (n=5), rarely (n=1), and never (n=1). For the statement “I experience anxiety or distress when I see a dirty toilet” responses were always (n=4), often (n=4), sometimes (n=2), rarely (n=2), and never (n=0). Four out of 16 participants (25%) reported having prior VR experience. Table 1 displays descriptive statistics for the main study variables. Correlations between primary variables of interest were examined and are presented in Table S1 in Multimedia Appendix 1. No unexpected associations were observed.

**Table 1.** Descriptive statistics for primary study variables in a single-arm, pretest-posttest pilot study of a single-session virtual reality public toilet exposure protocol in adults with subthreshold contamination concerns at Imperial College London (London, United Kingdom), February 2023-June 2023. Values are pooled across 5 imputed datasets.

Variable	Mean (SD)	95% CI
Baseline contamination score	5.75 (3.51)	4.03-7.47
Perceived OCD^a^ 1^b^	3.92 (1.07)	3.32-4.53
Perceived OCD 2^c^	3.49 (1.09)	2.88-4.09
Perceived OCD 3^d^	3.19 (0.91)	2.64-3.74
Presence 1^e^	4.62 (1.78)	3.75-5.5
Presence 2^f^	4.56 (1.59)	3.78-5.34
Plausibility 1^g^	4.19 (0.91)	3.74-4.63
Plausibility 2^h^	3.56 (1.03)	3.06-4.07
System Usability Scale (SUS)	69.50 (13.70)	62.79-76.21
Contamination score at follow-up	4.45 (2.37)	3.17-5.73

a OCD: obsessive-compulsive disorder.

b Perceived OCD 1: “I experience anxiety or distress when I see a dirty toilet.”

c Perceived OCD 2: “How often do you find yourself avoiding public toilets for fear or worry that they might be too dirty?”

d Perceived OCD 3: “How often do you find yourself unable to use public toilets for fear or worry that they might be too dirty?”

e Presence 1: “I had the sensation of being in the public toilet.”

f Presence 2: “I had the sensation that the scenario was really happening.”

g Plausibility 1: “Compared to a real public toilet, did you feel as uncomfortable and/or anxious in the virtual environment?”

h Plausibility 2: “Take a second to imagine that you are in a public toilet. Compared to this imagined public toilet, did you feel as uncomfortable and/or anxious in the virtual environment?”

### Symptom and Anxiety Outcomes

The Friedman test for negative affect indicated a statistically significant difference across session time points (*χ*²_3_=13.76; *P*=.003; W=0.29), with negative affect increasing after Exposure 1, returning toward baseline after the neutral break, and increasing again after Exposure 2. The repeated measures ANOVA for positive affect revealed a statistically significant difference during the VRET session (range across 5 imputed datasets: *F*_3, 45_=4.60-4.71; *P*=.006-.007; generalized eta squared=0.070-0.073), with positive affect decreasing from baseline to the end of Exposure 1 and then increasing from the break to the end of Exposure 2. For anxiety in the first VR exposure, the Friedman test indicated a statistically significant difference in SUDS scores across time points (*χ*²_3_=28.56; *P*<.001; W=0.60), with anxiety increasing during exposure and decreasing following virtual handwashing. For the second VR exposure, a repeated measures ANOVA again indicated a significant difference in SUDS scores across time points (Greenhouse-Geisser corrected: *F*_1.85, 27.81_=5.35; *P*=.01; generalized eta squared=0.058), with anxiety increasing during exposure and decreasing following virtual handwashing.

As an exploratory analysis, we examined whether baseline contamination severity (OCI-R contamination subscale) was associated with within-session distress during the VR protocol, indexed by the third SUDS during the first exposure trial (prior to virtual handwashing). Baseline contamination severity was not significantly correlated with peak distress (Spearman ρ=0.18; *P*=.51). Given the small sample size (n=16), the CI around this estimate is extremely wide and includes both strong positive and strong negative relationships; accordingly, no conclusions can be drawn from this analysis.

Figure 3 displays a graph of average anxiety levels over both VR exposures, including the 4 tasks that the participants experienced. Anxiety levels rose from baseline as participants interacted with the contaminated-looking VR environment and declined after exposure when they performed virtual handwashing. Highest levels of anxiety were reported when participants were engaging most directly with the contaminated environment (eg, touching the dirty-looking stall wall or toilet brush).

**Figure 4.**
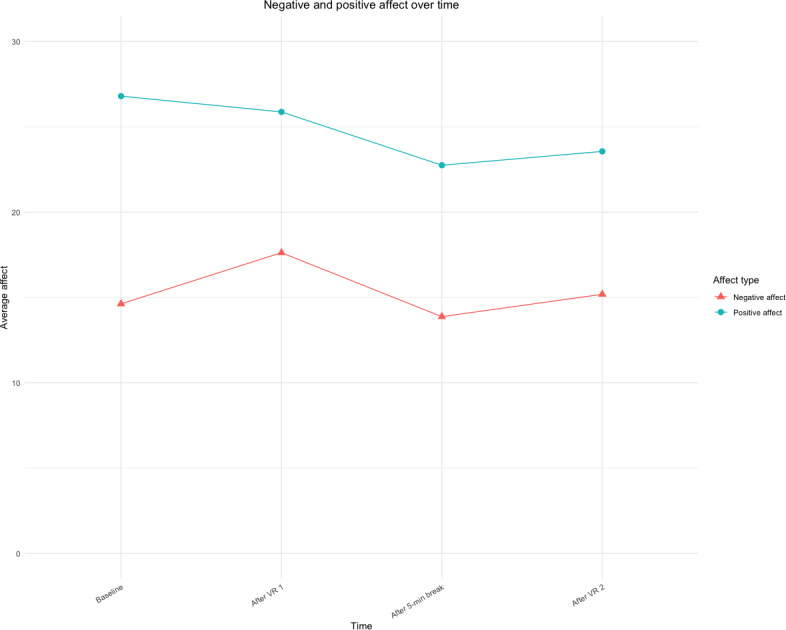
Mean positive affect and negative affect (Positive and Negative Affect Schedule [PANAS]) assessed at baseline, after Exposure 1, after the neutral break (5 minutes), and after Exposure 2 in a single-arm, pretest-posttest, single-session virtual reality exposure pilot study in adults with subthreshold contamination concerns at Imperial College London (London, United Kingdom), February-June 2023. VR: virtual reality.

Figure 4 illustrates average negative and positive affect over time, showing an increase in negative affect from baseline after the first VR exposure, a return to baseline following a 5-minute break, and another rise after the second VR exposure. Positive affect declined after the 5-minute break, with a slight decrease from baseline to the end of the first VR exposure, followed by a small increase from the break to the end of the second VR exposure.

To explore changes in contamination symptoms from baseline to 1-month follow-up, we conducted a paired-samples *t* test comparing OCI-R contamination scores at baseline (mean 5.75, SD 3.51) and at one-month follow-up (mean 4.45, SD 2.38). The results were not statistically significant (*t*_349.57_=1.54; *P*=.12; 95% CI –0.36 to 2.96), with an effect size of Hedges *g*=0.40. The large degrees of freedom reflect the multiple imputation pooling approach and the low fraction of missing information for this estimate. However, the CI included 0, indicating uncertainty in the magnitude of symptom change.

A complete-case sensitivity analysis was conducted restricted to the 10 participants with observed follow-up data. The complete-case paired *t* test yielded a mean difference of 0.40 (95% CI −0.96 to 1.76; *t*_9_= 0.67; *P*=.52; Hedges *g*=0.12), compared with the imputed estimate of 1.30 (95% CI −0.36 to 2.96; *t*_349.57_=1.54; *P*=.12 Hedges; *g*=0.40). The divergence between these estimates, particularly the reduction in effect size from Hedges *g*=0.40 to Hedges *g*=0.12, indicates that the imputed contamination finding is sensitive to the missingness assumption. The PANAS outcomes showed greater consistency between imputed and complete-case estimates (positive affect: Hedges *g*=0.83 vs 0.98; negative affect: Hedges *g*=−0.39 vs −0.39), suggesting the imputation performed more reliably for outcomes with lower follow-up missingness.

Separately, effect size calculations were conducted to describe the magnitude of observed changes in symptom severity and affect at 1-month follow-up. The estimated effect size for baseline to one-month follow-up contamination scores, as measured by the contamination OCI-R subscale, was moderate (Hedges *g*=0.40). However, this estimate should be interpreted cautiously, given the small sample size and uncontrolled single-arm design. For affect outcomes, a moderate negative effect size (Hedges *g*=–0.39; lower negative affect at follow-up) was found for negative affect, while a large positive effect size (Hedges *g*=0.83) was found for positive affect, suggesting potential change over time. However, these effect sizes are descriptive and do not establish clinical or causal effects in this pilot study.

### Participant Experience

To assess participants’ perception of the virtual environment, VR presence (sense of physical immersion and engagement) and plausibility were examined.

Participants varied in their sense of presence during the VR session. For the statement “I had the sensation of being in the public toilet,” most participants rated their sense of presence at the midpoint or above. One participant selected 1 (strongly disagree), one selected 2, 3 selected 3, one selected 4, 4 selected 5, 4 selected 6, and 2 selected 7 (strongly agree). For the statement “I had the sensation that the scenario was really happening,” one participant selected 1 (strongly disagree), none selected 2, 3 selected 3, 3 selected 4, 5 selected 5, 2 selected 6, and 2 selected 7 (strongly agree). Together these responses indicate that most participants reported at least a moderate sense of presence in the virtual environment.

Responses to the 2 plausibility items showed that participants generally found the VR environment comparable to or less anxiety-provoking than real and imagined scenarios. For the comparison with a real public toilet, no participant selected 1 (much more anxious in VR), one selected 2, 2 selected 3, 6 selected 4, and 7 selected 5 (much more anxious in the real toilet). For the comparison with an imagined public toilet, no participant selected 1, 3 selected 2, 4 selected 3, 6 selected 4, and 3 selected 5 (much more anxious in the imagined toilet).

Participants reported adequate acceptability for the VRET intervention. On the SUS, participants scored between 30.0 and 87.5, with a mean of 69.50 (SD 13.70; 95% CI 62.79-76.21), where a score of 68 is typically considered average and acceptable [55]. A majority found the system easy to use, usable without technical support, felt confident using the system, and thought that most people would learn to use the system quickly. A participant described using the intervention as “...an enjoyable, fun experience and a good way for exposure therapy without causing too much distress.” Another participant wrote, “...overall it was very good and well instructed. I can see how multiple sessions of this kind could help with this issue as it wouldn’t feel so daunting to enter contaminated places of this kind.” Complete participant feedback is listed in Table S2 in Multimedia Appendix 1.

Only 1 out of 16 (6.25%) participant reported VR sickness when asked directly at the end of the session. This reflects a global self-identification of sickness, which may differ from SSQ-measured symptom composites: the SSQ aggregates weighted ratings across multiple individual symptoms, including nausea, oculomotor discomfort, and disorientation, such that participants may endorse individual symptoms at meaningful levels without identifying themselves as experiencing VR sickness overall. SSQ scores are therefore reported separately as a more granular index of sickness-related symptoms. VR sickness scores were measured by the SSQ after each exposure. Disorientation was the highest reported symptom. Total VR sickness was much higher after the second (mean 105.89, SD 13.64; 95% CI 99.20-112.57) than after the first exposure (mean 25.25, SD 25.13; 95% CI 12.93-37.56). Total scores for VR sickness ranged from 0 to 78.54 for postfirst exposure and 78.54-130.90 for postsecond exposure.

Participants suggested several areas of improvement to the VR environment. One participant mentioned that, “...the avatar’s body blocked the view of some elements, so perhaps adjusting the dimensions of the avatar could enhance the viewing experience.” Participants also mentioned, “somehow trying to recreate the smell, though that might be quite gross,” and

..*.maybe if the environment had been changing more, like someone banging on the door or sounds of other people defecating, it would’ve felt more real. I think those are the kind of experiences that the VR might actually make me more resilient to.*

[Participant]

Participants frequently expressed a desire for changes in the avatar’s body dimensions (ie, the amount of body seen from the first-person avatar perspective) to allow for a clearer view inside the toilet bowl, as well as multisensory enhancements (eg, sounds and smells) to increase plausibility and immersion. Table S2 in Multimedia Appendix 1 includes a full list of participant feedback.

## Discussion

### Principal Results

This pilot study evaluated the feasibility and acceptability of a single-session VRET contamination exposure protocol, characterized within-session affective responding, and exploratorily assessed contamination symptom change at 1-month follow-up. Addressing these aims in turn: the VR protocol successfully elicited within-session anxiety during contamination exposure tasks, and affect fluctuated in patterns consistent with emotional responding during exposure. The intervention was feasible and acceptable, with participants rating usability as adequate and VR sickness not preventing session completion. However, a single session was not associated with statistically detectable contamination symptom reduction at 1-month follow-up.

### Comparison With Prior Work

Changes in anxiety across both exposures, increasing during interaction with the contaminated environment and decreasing following virtual hand-washing, demonstrated that the VR environment was capable of eliciting momentary anxiety. However, the postexposure anxiety decrease cannot be unambiguously attributed to extinction learning. A key design consideration concerns the inclusion of virtual hand-washing at the conclusion of each exposure trial. In standard ERP for contamination OCD, the therapeutic mechanism depends on preventing the compulsive response so that anxiety reduces without the reinforcing relief of completing a compulsion, thereby promoting extinction learning [56]. In the present protocol, virtual hand-washing was retained as an ecologically grounded feature reflecting normative post–toilet behavior, as washing hands after toilet use is typical in this context, and the study was not designed as a full ERP protocol. As a result, the observed postexposure anxiety decreases may equally reflect relief following completion of a compulsive washing behavior, which would be mechanistically opposite to the extinction learning process that ERP is designed to promote. This affects the degree to which the anxiety trajectory can be interpreted as consistent with exposure-based processes in the strict clinical sense [56], and future iterations should consider incorporating structured response prevention to test this more rigorously. Anxiety levels were slightly higher on average during the first exposure compared to the second. While this pattern could reflect habituation to the feared stimulus, it may also reflect reduced novelty, increased task familiarity, or boredom effects, given that participants had just completed an identical trial. The study design does not permit distinguishing among these explanations, and this should be considered when interpreting the between-trial difference. The limited nature of the intervention may not have been sufficient to establish habituation or for habituation to become ingrained in real-life scenarios [56], and further research is needed to determine therapeutic benefits. This is consistent with Inozu et al [35], who found that significant reductions in contamination-related anxiety required a minimum of 3 VR-ERP sessions delivered across several weeks, suggesting that a single session is unlikely to produce lasting symptom change. Fajnerová et al [34] similarly found that overall anxiety levels did not significantly increase in patients with OCD following a single VR exposure session, raising questions about the dose of exposure required to produce meaningful affective change.

This is further supported by the observed changes in positive and negative affect. Figure 4 shows negative affect rising from baseline after the first VR exposure, returning to baseline following the 5-minute break, and increasing again after the second VR exposure. These fluctuations are consistent with within-session affective responding patterns described in the exposure therapy literature, in which negative affect is expected to increase during contact with feared stimuli and decrease during recovery intervals [56]. Positive affect decreased after the 5-minute break, with a slight drop from baseline to the end of the first VR exposure and a small increase from the break to the end of the second exposure. This pattern may reflect the novelty of the intervention and a sense of relief at the session’s conclusion, though direct evidence for these explanations is not available in the present data.

Most participants felt a strong sense of VR presence but did not find the environment overly anxiety-provoking compared to real or imagined public toilet scenarios. The adequate usability profile and low distress burden observed here also raise the possibility of a role for VRET as a stepping stone within a treatment hierarchy, positioned between imaginal exercises and in vivo exposure, as suggested by comparisons of VRET and in vivo approaches [41], though this remains speculative and untested in the present design. VR offers a more realistic and immersive context than imaginal exposure while remaining more controllable and safer than in vivo contact with feared stimuli [41], potentially allowing individuals to build tolerance and self-efficacy before progressing to real-world exposures. However, this remains speculative on the basis of the present data, and direct evidence for the stepping stone hypothesis would require studies comparing outcomes in samples who receive VRET prior to in vivo ERP against those who proceed to in vivo ERP directly. While the VR environment successfully elicited anxiety, the single-session design may have limited the opportunity for habituation, which has been identified as a key process in exposure-based symptom reduction [56]. These results support the feasibility of delivering a standardized contamination-focused VR exposure protocol and provide acceptability and within-session responding data that can inform the design of future multi-session and controlled trials. Participants rated the system’s usability as adequate, clearing the conventional threshold for acceptable usability by a modest margin [55], felt confident using the intervention, and found it manageable without technical support. These findings indicate that the protocol meets a basic threshold for usability, though there remains meaningful room for improvement, as also reflected in participants’ qualitative feedback on avatar dimensions, multisensory limitations, and environmental realism. These findings are consistent with broader interest in scalable digital health interventions that can be delivered with reduced clinician involvement [22,43], though whether this protocol can be meaningfully deployed as homework or with minimal supervision requires direct evaluation. Usability findings also suggest that prior VR experience may not be a prerequisite for engaging with the protocol, though replication in samples with greater age diversity and varying levels of technological familiarity is needed.

Though only one participant identified themselves as experiencing VR sickness when asked directly, SSQ scores indicated that sickness-related symptoms were present across the sample, particularly after the second exposure. This discrepancy reflects the distinction between global self-identification of sickness and a multi-item symptom composite, as discussed in the Results. VR sickness did not prevent session completion for any participant, and the limited avatar movement likely contributed to the relatively contained symptom profile observed after the first exposure [57]. Disorientation was the most commonly endorsed symptom cluster, more so than nausea or oculomotor symptoms. This may be due to technical issues like latency, inconsistent frame rates, or unnatural field of view and depth perception [57]. It could also result from participant fatigue (spending too long in VR without breaks to reset proprioception) or inadequate embodiment (not feeling connected to the avatar as their own body), though these remain speculative explanations in the absence of direct measurement. The substantially higher SSQ scores after the second exposure suggest that symptom burden increased over the course of the session, which should be considered in future protocol design.

Our VR sickness scores after one exposure (mean 25.25, SD 25.13) were comparable to those of the healthy control group in Fajnerová et al [34], who reported median scores of 21.50 (SD 58.08). This is likely because the participants in this study had symptoms below the clinical threshold, making them more similar to healthy controls than to the diagnosed OCD group in Fajnerová et al [34]. However, the teleportation movement mechanic used in the VR environment of Fajnerová et al [34], which was not used in this study, likely contributed to higher VR sickness scores.

The present pilot study makes several specific contributions to the VR exposure literature. First, it extends prior work by evaluating a standardized single-session contamination-focused VR exposure protocol in adults with elevated contamination concerns who did not meet diagnostic criteria for OCD, a population underrepresented in VRET research despite clinical relevance for scalable early intervention [37,40], and establishes that the protocol is feasible and acceptable in this group specifically. Second, the study demonstrates that the VR environment reliably elicited within-session anxiety across both exposure trials in a subthreshold sample. Because fear activation during exposure is considered a prerequisite for therapeutic change [56], this finding is a meaningful indicator that the environment functions as intended and supports progression to efficacy testing. Third, the finding that a single session was not associated with symptom change at 1-month follow-up, while exploratory, directly informs future protocol development by ruling out single-session dosing as a sufficient therapeutic dose for this population and pointing toward multisession designs. Finally, adequate usability ratings and VR sickness levels that did not prevent session completion suggest the protocol could potentially be delivered without live clinical supervision, with implications for scalable implementation among individuals with elevated contamination concerns who have not yet sought formal treatment.

### Limitations

This pilot study is not without limitations. The study’s sample size was limited, which could have potentially diminished its generalizability. In addition, ethnicity was not collected, which limits characterization of sample diversity and may further constrain generalizability. Accordingly, the nonsignificant symptom change finding should not be interpreted as evidence of a true null effect. The observed effect size was moderate, and a moderate effect in a sample of this size is consistent with insufficient statistical power to detect a real difference. The direction of the effect, suggesting a trend toward symptom reduction, provides a preliminary basis for sample size estimation in future adequately powered trials. Future studies targeting a similar population should be powered to detect effects in the small-to-moderate range. Even so, the study offers valuable preliminary data that will inform future research endeavors. The broad range of contamination scores (as measured by the contamination subscale on the OCI-R) represents a full spectrum of contamination-related anxiety. However, the inclusion of participants with relatively low contamination scores could have diluted the observed pre-post changes, potentially masking significant findings or underestimating the true magnitude of change among individuals with higher baseline symptoms. Nevertheless, a sensitivity analysis for contamination scores was not feasible due to the small sample size. The follow-up noncompletion rate of 37.5% (6/16) participants means that the primary symptom outcome was missing for a substantial proportion of the sample. Although multiple imputation with chained equations was applied under a missing-at-random assumption, this assumption may not hold because participants who did not return for follow-up may differ systematically from those who did in ways not fully captured by observed variables; for example, in terms of engagement with the intervention or symptom trajectory. Imputing 37.5% of follow-up data in a sample of n=16 is inherently substantial, and pooled estimates for the contamination outcome should be interpreted with particular caution. A complete-case sensitivity analysis is reported in the Results section. Briefly, the primary symptom finding was sensitive to the missingness assumption, with the complete-case and imputed effect size estimates diverging substantially, indicating that the imputed contamination finding should not be taken as a reliable estimate of the true effect. PANAS outcomes showed greater consistency between the 2 estimation approaches, suggesting the imputation performed more reliably for outcomes with lower follow-up missingness. Multiple imputation was conducted with m=5 imputations, consistent with common practice in small-sample pilot work, though this may introduce additional Monte Carlo error in pooled estimates. It is also worth noting that compensation was not contingent on completion of the one-month follow-up survey, which may have contributed to the 37.5% follow-up dropout rate and the resulting missingness in the primary symptom outcome data.

Another constraint is the lower internal consistency observed for the OCI-R subscale at the one-month follow-up compared to baseline. Since the OCI-R demonstrated strong reliability at baseline, this decline is unlikely due to flaws in the measure itself. Indeed, repeated exposure to tests can affect participant performance, and sample attrition can impact the consistency of measurements, potentially distorting reliability estimates [58]. Furthermore, while measuring contamination symptoms using the OCI-R subscale at the one-month follow-up may present limitations for detecting change following a single-session intervention, prior research has demonstrated that even single-session VRET interventions can produce measurable effects at one month or later [59,60]. Participant bias is another potential limitation, given the novel nature of the technology and the recruitment method. Specifically, self-selection bias may have led participants to be more motivated to address their contamination-related fears, possibly increasing the perceived benefit of the intervention. Additionally, individuals drawn to the pilot study may have had a preexisting interest in VR technology, which could have influenced engagement and overall perception of the intervention, regardless of its direct impact on symptoms.

Furthermore, participants were recruited via Instagram advertisements and campus posters at a single UK university, which may have introduced a bias toward individuals with above-average technology familiarity and engagement with digital platforms. This is directly relevant to the interpretation of usability and acceptability findings: the usability scores observed in a potentially technology-comfortable sample may not generalize to older or less technology-familiar populations who would be among the primary beneficiaries of a scalable, low-barrier intervention. Usability and acceptability findings should therefore be interpreted with this recruitment context in mind.

It should also be noted that because Exposure 1 and Exposure 2 SUDS data were analyzed using different test families, reflecting differences in distributional assumptions, formal statistical comparison of results between trials is not straightforward. Between-trial differences should therefore be interpreted descriptively rather than inferentially.

This study used a single-group, nonrandomized design without a comparison condition, which limits causal inference. As a result, changes observed from baseline to follow-up cannot be attributed definitively to the VR exposure protocol and may reflect nonspecific factors such as assessment effects, expectancy effects, or the novelty of engaging with VR. In addition, symptom change at follow-up may be influenced by regression to the mean, particularly given the subthreshold nature of the sample and the single-session format. Accordingly, symptom outcomes should be interpreted as preliminary and exploratory, and future studies should include randomized controlled designs with appropriate comparator conditions to evaluate efficacy. Bayesian approaches may be useful in future larger-scale studies to quantify evidence for change versus no change, particularly when effects are expected to be small.

Although exposure-based treatment for OCD is typically delivered across multiple sessions [14], the goal of the present pilot was not to evaluate clinical efficacy but to establish whether a standardized single-session VR protocol was feasible, acceptable, and capable of eliciting distress during exposure tasks. The single-session design was selected to reduce participant burden and to support scalable early-stage testing of the protocol, particularly given the subthreshold nature of the sample and the study’s emphasis on user experience. We view this study as a first step in developing a replicable VR exposure procedure, with future work needed to evaluate multisession dosing and to test clinical outcomes using randomized designs and diagnostically confirmed samples.

Despite these limitations, our pilot study has several strengths, as it offers a comprehensive analysis by examining both the program’s usability and its acceptability as an intervention. Evaluating usability ensures that users can navigate the program effectively, which is crucial for maintaining engagement and optimizing the user experience [55]. In contrast, assessing acceptability illuminates how well the intervention is received by users, providing preliminary information relevant to future integration into clinical practice and informing efforts to optimize treatment engagement. In addition, by incorporating both quantitative study design elements and open-ended questions, we gain a nuanced, multifaceted understanding of critical factors, such as participant perceptions of usability, intervention acceptability, and subjective experiences during VR exposure tasks. Furthermore, rather than focusing solely on OCD symptom reduction, we explore additional dimensions such as anxiety, as well as positive and negative affect. This broader evaluation provides insight into the emotional processes during exposure, such as the temporary increase in negative affect and recovery during the break, supporting the VRET’s ability to provoke and modulate anxiety.

These findings carry specific implications for future research and clinical translation. The adequate usability profile and tolerability observed here suggest that self-administered or minimally supervised delivery may be worth evaluating in future trials [22,43], with particular relevance for scalable early intervention among individuals with elevated contamination concerns who have not yet accessed formal treatment, including those in settings with limited access to specialist OCD care. Future research should prioritize multisession designs [56] with larger, diagnostically confirmed samples and appropriate control conditions to evaluate therapeutic efficacy, with attention to protocol parameters such as response prevention structure and exposure dose. Refinement of the virtual environment, including enhanced contamination cues and multi-sensory features such as spatial audio, will support development of a clinically optimized protocol. This line of work holds promise as a pathway to reducing barriers to early intervention in a population that remains largely underserved by current treatment pathways.

### Conclusions

This study is among the first to systematically evaluate a standardized single-session contamination-focused VR exposure protocol in adults with elevated contamination concerns who did not meet diagnostic criteria for OCD, a subthreshold population largely underrepresented in prior VRET research, which has predominantly focused on clinically diagnosed samples and multisession protocols [27,28,38,39]. By directly targeting this underrepresented group, the study provides feasibility and acceptability benchmarks and within-session anxiety data not available from existing literature focused on clinical samples [37,40]. A single session was not associated with significant symptom change, and the effect size estimate for contamination symptom reduction was sensitive to the missingness assumption, indicating that a single session is unlikely to constitute an adequate therapeutic dose for this population and underscoring the need for adequately powered multisession designs in future efficacy trials. Nevertheless, the protocol reliably elicited within-session anxiety during exposure tasks, was rated as usable and manageable by participants, and was associated with VR sickness that did not prevent session completion, indicating that the protocol functions as intended in this population and supports progression toward efficacy testing.

## Supplementary material

10.2196/78169 Multimedia Appendix 1 Supplementary tables showing correlations between main study variables (Table S1) and participant open-ended feedback on the virtual reality experience (Table S2).

## Abbreviations

ERP: exposure and response prevention
OCD: obsessive-compulsive disorder
OCI-R: Obsessive-Compulsive Inventory–Revised
PANAS: Positive and Negative Affect Schedule
SSQ: Simulator Sickness Questionnaire
SUDS: Subjective Units of Distress Scale
SUS: System Usability Scale
VR: virtual reality
VRET: virtual reality exposure–based therapy
XR: extended reality
