# Supplementary material for: Virtual Reality–Delivered Exposure for Contamination Concerns in Adults With Obsessive-Compulsive Symptoms: Single-Arm Pilot Study
Source: JMIR Serious Games. 2026 Jun 23;14:e78169. doi: 10.2196/78169 (PMC13290166; doi:10.2196/78169)
Supplement: Multimedia Appendix 1 [file games-v14-e78169-s001.docx]

**Table S1**

### *Correlations Between Main Study Variables*

|  | 1 | 2 | 3 | 4 | 5 | 6 | 7 | 8 | 9 | 10 | 11 | 12 13 |
| --- | --- | --- | --- | --- | --- | --- | --- | --- | --- | --- | --- | --- |
| 1. Perceived OCD symptoms 1 |  |  |  |  |  |  |  |  |  |  |  |  |
| 2. Perceived OCD symptoms 2 | .40 |  |  |  |  |  |  |  |  |  |  |  |
| 3. Perceived OCD symptoms 3 | .30 | .39 |  |  |  |  |  |  |  |  |  |  |
| 4. Baseline contamination symptoms | .19 | .34 | .12 |  |  |  |  |  |  |  |  |  |
| 5. Average anxiety in exposure 1 (SUDS) | .53 | .34 | .40 | .06 |  |  |  |  |  |  |  |  |
| 6. Average anxiety in exposure 2 (SUDS) | .43 | .15 | .36 | -.04 | .91*** |  |  |  |  |  |  |  |
| 7. Baseline positive affect | -.25 | -.14 | -.48 | .03 | -.23 | -.21 |  |  |  |  |  |  |
| 8. Baseline negative affect | -.08 | .03 | .04 | .57 | .32 | .36 | .17 |  |  |  |  |  |
| 9. VR sickness after exposure 1 | .18 | .25 | .18 | .01 | .41 | .23 | -.43 | .10 |  |  |  |  |
| 10. VR sickness after exposure 2 | .56 | .50 | .34 | .47 | .52 | .36 | -.09 | .18 | .45 |  |  |  |
| 11. System usability scale | .01 | -.19 | -.12 | .17 | -.03 | .16 | .21 | .07 | -.66 | -.29 |  |  |
| 12. Post-intervention contamination symptoms | .20 | .04 | .26 | .46 | -.07 | -.03 | -.11 | .07 | -.37 | .17 | .32 |  |
| 13. Post-intervention positive affect | .05 | -.36 | -.21 | -.17 | -.16 | -.10 | .34 | -.19 | -.06 | -.09 | .15 | .00 |
| 14. Post-intervention negative affect | .06 | .01 | .37 | .13 | .60 | .68 | -.12 | .61 | .09 | .21 | .07 | .28 -.21 |

*Note.* Post-imputation pooled Spearman’s *ρ* shown with False Discovery Rate correction applied.

**p* < .05. ***p* < .01. ****p* < .001.

**Table S2**

### *Feedback and acceptability open-ended responses*

| **Question** | **Participant Response** |
| --- | --- |
| **How did you feel while you were taking part in the virtual reality experience?** | Intrigued about what would happen next, yet also anxious.  The initial ’unknown’ experience made me feel uncomfortable, however knowing what was coming, in the second environment, made me feel more anxious, where I thought that it would make me feel less anxious, as I knew what I would be faced with.  Intrigued about what would happen next, yet also anxious. uncomfortable, disgusted  I felt disgusted by the surroundings, especially the cubicle Excited at first and then a little nervous as we put the headset on. Overall quite uncomfortable  I felt quite unprepared and awkward because I didn’t know what to expect and I couldn’t see what my reactions were like. I also felt quite determined to get through it even it seemed daunting.  I felt nervous, then I felt gross when I got in the bathroom  anxious due to unfamiliarity and unease around public toilets, slightly overwhelmed and at first dizzy/uncoordinated I feel it is really realistic and it makes me feel surreal |

*Continued on next page*

Table S2 – *Continued from previous page*

| **Question** | **Participant Response** |
| --- | --- |
|  | interested and curious  normal, then grossed out and with a slight eye strain curious but not too worried  I felt nervous about the study itself and trying to figure out what I was supposed to be feeling. I did also feel grossed out by the environment, but it didn’t feel real enough to make me feel like I was actually there. But it was still a bit oppressive, and together with the motion sickness of the VR headset, I did feel quite uncomfortable.  Quite interesting in general, this is the first time I used a VR set. |
| What did you think when you saw the con- taminated environment? | It reminded me of previous experiences I have had in this scenario.  it was not safe  I was wondering as to how an environment could reach that point - and reminded me of an experience that I have had recently, where I was in a similar situation - and upon reflection, I can now see myself doing things in my real-life environment that I didn’t notice at the time, however I avoided them/did them differently, at the time.  I thought it was disgusting and wanted to leave as soon as possible That it was very dirty and I’d need to not touch anything.  It was very realistic, I didn’t want to open the cubicle door (details like loo roll on the floor and the gross full bin made it more realistic)  I was quite surprised about the realness of it in general because it matched the kind of environment I had pictured better than I thought it would. I also thought that it was really unpleasant in general.  that i wanted to leave straight away, i would’ve rather gone to pee outside somewhere in a park  I thought it was a particularly disgusting bathroom. I would have immediately left if I wasn’t absolutely desperate. I noticed the trash on the floor and grimy toilet seat the most. I also noticed the Pepe on the door and thought that was fun.  that I wouldn’t want to be there  I feel I want to finish everything really quick without touching anything and leave I want to escape  disgust  It looked typical based on the outside environment It’s normal in public place  I didn’t like it, I could imagine one in real life |
| How did you feel when you saw the con- taminated environment? | It made me feel anxious, reluctant to use, on edge.  slightly nervous  The initial ’unknown’ experience made me feel uncomfortable, however knowing what was coming, in the second environment, made me feel more anxious, where I thought that it would make me feel less anxious, as I knew what I would be faced with.  I felt disappointed but not surprised |

*Continued on next page*

Table S2 – *Continued from previous page*

| **Question** | **Participant Response** |
| --- | --- |
|  | I felt nervous and contaminated I felt sweaty and dirty.  tense  Slightly anxious and ill at ease  Feeling rushed to complete the stuff as soon as possible  I felt disgusted and repulsed, and quite uncomfortable. I just really wanted to leave.  I felt gross and dirty  that I wouldn’t want to be there  anxious and distressed, not wanting to touch anything  I didn’t feel distressed but I felt a bit uneasy. I also felt quite unsafe and exposed.  At first a little disgust, especially when wiping the toilet clean is not an option, but otherwise it is alright. I feel quite a bit stressed and upset. |
| In your opinion, is there anything that could have been done differently to improve the software or create a better experience (in any aspect)? | Adding smell and sound as these could alter the experience significantly  Adding the smell to make it more realistic. Make participants sit on a real toilet. smell or touch elements  Different headset that can change the focus for people with glasses - could have taken off my glasses, and still been able to see. High resolution, higher framerate, more comfortable headset to wear  Maybe having real objects to touch when going through the motions of the simulation More freedom in choosing what to do.  I would maybe improve the visual environment’s quality  Smell should be simulated simultaneously when running the VR application; The VR scene is not that horrible enough.  Some of the graphics were a little blurry and the avatar’s body blocked the view of some elements so perhaps adjusting the dimensions of the avatar could enhance the viewing experience.  Somehow trying to recreate the smell, though that might be quite gross. Also, in the cubicle scene, the view of the actual toilet, where the water is, was blocked by the avatars body and I couldn’t maneuver past it in any way. I think I could see the bowl better, and if it was perhaps blocked and smeared with poop, and the floor was wet, I would’ve felt much more distressed. Also maybe if the environment had been changing more, like someone banging on the door or sounds of other people defecating, it would’ve felt more real. I think those are the kind of experiences that the VR might actually make me more resilient too.  The body can be smaller, giving a better field of view. Maybe adding the actual walking animations instead of teleporting around. The flesh was luminous and the body obscured a lot of things (e.g. toilet brush).  calibrate the avatar better so it won’t block the scenario |

*Continued on next page*

Table S2 – *Continued from previous page*

| **Question** | **Participant Response** |
| --- | --- |
|  | maybe more realism, such as feeling a wall or acting it out, maybe smelling the environment  smaller avatar body so the field of vision wasn’t blocked |
| Do you have any additional comments about the virtual reality program and/or your experience with it? | It was an enjoyable, fun experience and a good way for exposure therapy without causing too much distress.  Difficult to do so with glasses, however once that issue had been sorted, the overall environment was ’enjoyable’ The environment wasn’t very detailed.  It would be interesting to do different scenarios, such as using a urinal rather than a stall. Also experimenting with space and manipulating other senses, such as smell and sound  I felt uncomfortable performing the actions the avatar was performing (i.e. acting them out in the real world whilst wearing VR) but maybe that could have made it more realistic.  Adding in smell or touch elements to the VR experience could help you feel more immersed, but overall it was very good and well instructed. I can see how multiple sessions of this kind could help with this issue as it wouldn’t feel so daunting to enter contaminated places of this kind.  Generally interesting  I thought it was pretty exciting realistic looking, would be much more interactive if I was able to perform the actual tasks the avatar did automatically such as touch the wall or sit down without a change in the perspective.  it was interesting since it was my first time using VR  I feel like the lack of actually moving and feeling things like the toilet brush or toilet seat took me out of the experience of actually being there  if it was more realistic it would have felt more immersive but also cause more anxiety so i think it was done well I think it is good in general.  The scene was not very delicate and detailed but it was really realistic VR is really interesting. It was a nice experience.  was great  I wonder if there was less of a virtual barrier, (meaning if I actually felt as if it was a real toilet) if my anxiety and concerns would feel more real |
